# Buparvaquone Nanostructured Lipid Carrier: Development of an Affordable Delivery System for the Treatment of Leishmaniases

**DOI:** 10.1155/2017/9781603

**Published:** 2017-02-01

**Authors:** Lis Marie Monteiro, Raimar Löbenberg, Paulo Cesar Cotrim, Gabriel Lima Barros de Araujo, Nádia Bou-Chacra

**Affiliations:** ^1^Department of Pharmacy, Faculty of Pharmaceutical Sciences, University of São Paulo, Professor Lineu Prestes Av 580, Cidade Universitária, 05508-000 São Paulo, SP, Brazil; ^2^Faculty of Pharmacy and Pharmaceutical Sciences, University of Alberta, 8613 114th St NW, Edmonton, AB, Canada T6G 2H7; ^3^Seroepidemiology, Cellular and Molecular Immunology Laboratory, Institute of Tropical Medicine, University of São Paulo, Dr. Enéas de Carvalho Aguiar 470, Jardim América, 05403-000 São Paulo, SP, Brazil

## Abstract

Buparvaquone (BPQ), a veterinary drug, was formulated as nanostructured lipid carriers (NLC) for leishmaniases treatment. The formulation design addressed poor water solubility of BPQ and lack of human drug delivery system. The DSC/TG and microscopy methods were used for solid lipids screening. Softisan® 154 showed highest BPQ solubility in both methods. The BPQ solubility in liquid lipids using HPLC revealed Miglyol® 812 as the best option. Response surface methodology (RSM) was used to identify the optimal Softisan154 : Miglyol 812 ratios (7 : 10 to 2 : 1) and Kolliphor® P188 and Tween® 80 concentration (>3.0% w/w) aiming for *z*-average in the range of 100–300 nm for macrophage delivery. The NLC obtained by high-pressure homogenization showed low *z*-averages (<350 nm), polydispersity (<0.3), and encapsulation efficiency close to 100%. DSC/TG and microscopy in combination proved to be a powerful tool to select the solid lipid. The relationship among the variables, demonstrated by a linear mathematical model using RSM, allowed generating a design space. This design space showed the limits in which changes in the variables influenced the *z*-average. Therefore, these drug delivery systems have the potential to improve the availability of affordable medicines due to the low cost of raw materials, using well established, reliable, and feasible scale-up technology.

## 1. Introduction

Over the past decades, improper prescription and lack of patient compliance, due to severe toxicity of conventional medicines, have caused the development of widespread parasite resistance in leishmaniases. Also, no new drug substances have been introduced in the therapy, and there are no effective vaccines available to prevent or heal this neglected tropical disease [1, 2]. Buparvaquone (BPQ) is used in the treatment of blood parasites in cattle and showed promising antileishmanial in vitro activity. Even though there is a formulation for veterinary use, no suitable pharmaceutical dosage form for a human is available. Also, the low water solubility of BPQ limits its bioavailability, which is the main challenge for developing a drug delivery system for enhancing its therapeutic efficacy.

The development of lipid nanoparticles is one of the most promising alternatives to overcome this limitation. Such delivery systems have the ability to overcome biological barriers, increase therapeutic efficacy, reduce toxicity, and allow drug release at the specific site [3, 4]. Moreover, these colloidal carriers are quickly taken up by cells from the mononuclear phagocyte system [5], which are the host cells of the leishmaniases parasite infection. This mechanism makes nanoparticles an attractive vector for passive targeting of antileishmanial drug substances [6]. Therefore, lipid-based nanoparticles represent an opportunity to improve the leishmaniases therapy.

Recently, biocompatible lipids have attracted attention for development of delivery systems for poorly soluble drugs [7]. Lipid-based nanoparticles include nanoemulsions (NE), solid lipid nanoparticles (SLN), and nanostructured lipid carriers (NLC). The NE present some advantages; they can be used in various dosage foam such as creams, liquids, sprays, and foams. However, the main challenges are the low stability of the systems, use of a large concentration of surfactant for stabilizing the nanodroplets, and limited solubilizing capacity for high-melting point substances [8].

SLN present disadvantages, such as low drug loading capacity and drug expulsion during storage. The low ordered lipid matrix tends to be reorganized to highly ordered structure. As a consequence, the perfect crystal lattice cannot accommodate the drug [9]. The addition of a liquid phase can disorder the lipid matrix, which avoids lipid polymorphism and leads to enhanced drug loading capacity [10].

NLC have been introduced as an alternative to traditional colloidal carriers such as liposomes and polymeric nanoparticles due to the lower cost of raw materials, in comparison with phospholipids used to prepare liposomes. These NLC can provide formulations with higher physical stability, high encapsulation efficiency, and feasible industrial scale-up [11]. Furthermore, the NCL combine the advantages of polymeric nanoparticles due to the presence of a solid matrix, which can protect chemically unstable drugs and allow controlled release. As well, NLC bear the advantage of low in vivo toxic, owing to the use of biocompatible and biodegradable materials [11].

Recently, the formulation design of salicylic acid and lidocaine-prilocaine NLC, using the design of experiments (DoE), was reported to address the low water solubility of these drugs [12, 13]. The DoE is superior to the traditional change-one-at-a-time approach because it is a systematic multivariate method. This powerful statistical tool can be used to create a design space aiming to achieve an in-depth knowledge of the formulation and process understanding. Design space is defined as the multidimensional combination and interaction of input variables (e.g., material attributes) and process parameters that have been demonstrated to provide assurance of quality [14].

Considering the leishmaniases therapeutic scenario, new drug substances and affordable drug delivery systems are urgently needed. Therefore, BPQ-NLC are promising formulations to achieve high therapeutic efficacy and safety for leishmaniases treatment. This work is focused on the comparison of two methods for BPQ solid lipid solubility studies (DSC/TG and microscopy) in the preformulation stage, and development and optimization of BPQ-NLC, using central composite experimental design.

## 2. Materials and Methods

### 2.1. Chemicals

BPQ was purchased from Uniwise (China). Softisan 154, Dynasan® P60, Dynasan 118, and Witepsol® E85 were kindly donated by CREMER Oleo Division (Germany). Gelucire® 44/13, Compritol® 888, Gelucire 50/14, and Precirol® ATO 5 were kindly donated by Gattefossé (France). Sterotex® HM, oleic acid, glyceryl monocaprylate, and medium chain triglycerides (MCT) were kindly donated by Abitec (USA). Super refined cottonseed, safflower, corn, olive, soybean, and sesame oils were kindly donated by Croda (UK). Kolliphor P188 was acquired from BASF (Germany) and Tween 80 from Sigma-Aldrich (Spain). Purified water was of Milli-Q quality (Millipore, USA). All other reagents were at least of analytical grade and were used without further purification.

### 2.2. Selection of Liquid and Solid Lipids for BPQ-NLCs Preparation

Regarding the liquid lipids, BPQ in excess was added to the liquid lipid and placed in orbital shaker at 25°C. After 24 hours, aliquots were filtered through a PVDF membrane of 0.22 *μ*m pore size and were injected into HPLC to determine the amount of solubilized BPQ, using the method described below.

BPQ solubility in solid lipids was evaluated according to Kasongo et al. [15]. Briefly, buparvaquone was added to the solid lipid in a concentration of 0.001% (w/w); this preparation was heated 10°C above the melting point of each lipid, under constant shaking for 24 hours. Aliquots were taken and observed by optical microscope; the BPQ concentration was adjusted until crystals observation. The results were ranked by the amount of crystals, the higher position in the rank, and the lower amount of crystals was observed.

Drug solubility and crystallization behavior in solid lipids were carried out by using DSC/TG. The BPQ thermal behavior and solid lipids and 1 : 1 physical mixtures (PM) were characterized in a DSC 4,000 Perkin Elmer cell (Perkin Elmer Corp., Norwalk, CT, USA), under a dynamic N_2_ atmosphere (50 mL·min^−1^), using sealed aluminum capsules with about 2 mg of samples. DSC curves were obtained at a heating rate of 10°C min^−1^ in the temperature range from 25 to 290°C. An empty sealed pan was used as a reference.

TG/DTG curves were obtained with a thermobalance model Exstar-7200 (Hitachi High-Tech Science Corporation, Tokyo, Japan) in the temperature range 25–600°C using platinum crucibles DSC Exstar-7020 (Hitachi High-Tech Science Corporation, Tokyo, Japan).

The onset temperature, maximum peak in the melting range (*T*_peak_), and melting enthalpy (Δ*H*_m_) were calculated using the software provided by PerkinElmer. The crystallinity indexes (CI) of BPQ were calculated in percentage according to the following equation [16–19]:

(1) $$\begin{array}{c} CI\;\left(\;\%\;\right)=\frac{\Delta H\;\;BPQ\;\;PM*D}{\Delta H\;\;BPQ\;\;100\%}*100, \end{array}$$

where Δ*H* BPQ PM is the enthalpy of fusion (J·mg^−1^) of BPQ in the binary physical mixture of BPQ and solid lipid. Δ*H* BPQ 100% is the enthalpy of fusion (J·mg^−1^) of pure drug. *D* is the dilution of BPQ in the PM, for example, 1 : 1: dilution of 2.

### 2.3. Preparation of BPQ-NLC

After lipids selection, a mixture of solid (SL) and liquid (LL) lipids was tested in different ratios of SL : LL, from 1.5 (3 : 2 SL : LL) to 4 (4 : 1). The melting point of the mixture was determined by DSC as described above. The optical homogeneity was accessed by smearing the mixture in glass slides and observation of liquid drops or solid crumbs. The homogeneous mixtures of melting point above 40°C were selected for BPQ-NLC preparation.

The NLC preparation was performed as proposed by Muchow et al. [20] by dispersing the lipid phase in the aqueous phase. The system was mixed by a mechanical homogenizer at 8000 rpm (Ultraturrax, IKA) for 5 min. The preemulsion was passed through a temperature-controlled high-pressure homogenizer (Nano DeBEE, BEE International), 60 bar and five cycles.

### 2.4. NLC Formulation and Optimization by Response Surface Design

A three-factor (2^3^) central composite design was employed for optimization of the nanostructured lipid carriers. The study was performed to evaluate the influence of the following independent variables: solid and liquid lipids ratio (SL : LL) and surfactants concentrations (independent variables) in the *z*-average (response or dependent variable). The response surface method was applied to define the optimal conditions of the process and identification of relevant variables and their working range. The SL : LL range was 1.5 (3 : 2) (w/w) to 4.0 (4 : 1) (w/w); the surfactants concentrations were from 1.0 to 3.0% (w/w). Minitab® 17 software was used to generate the design matrix and data analyses.

The desirability method was used for the optimization process. The desirability function is based on the optimization techniques developed by Derringer and Suich [21]. In this approach, each response *Yi* is first converted into a *DY* of each desirability function ranging between 0 and 1 (0 ≤ *di* ≤ 1), where *D*(*Y*) = 0 represents an entirely undesirable value *Yi*, while *D*(*Yi*) = 1 represents a completely desirable or ideal response value. The *DI* individual values are then combined using a geometric mean to give a general desirability function *D* [22].

### 2.5. Determination of *z*-Average, Polydispersity Index, and Zeta Potential

The *z*-average of the nanostructured lipid carriers was determined immediately after high-pressure homogenization and was assessed by photon correlation spectroscopy (PCS) using Zetasizer ZS90 (Malvern Instruments, Malvern, UK) at 25°C and 90° (*n* = 10). The PCS technique calculates the *z*-average as light-weighted intensity and polydispersity index (PDI) as a measure for the width of the *z*-average distribution. The equipment was also used to measure the zeta potential (ZP). ZP measurements were carried out in purified water with a conductivity adjusted to 50 *μ*S·cm^−1^ by the addition of NaCl 0.1% (w/v), to avoid ZP fluctuations caused by the difference in conductivity (*n* = 3). The pH was adjusted to 6.5 ± 0.2 by the addition of 0.01 N HCl or 0.01 N NaOH solution.

### 2.6. Determination of BPQ Solubility in Liquid Lipids and Encapsulation Efficiency

The method described by Venkatesh et al. [23] was used as a basis for quantification method for solubility in liquid lipids and encapsulation efficiency: in brief, C4 250 mm × 4.6 mm ID, 5 *μ*m particle size column; 1% glacial acetic acid in water, acetonitrile, and methanol 30 : 60 : 10 (v/v/v) mobile phase; *λ* = 251 nm; 50 *μ*L injection volume; 35°C column temperature. The final conditions were as follows: column Waters Xterra, C8 100 × 4.6 mm, 3.5 *μ*M particle size; a mobile phase of 1% glacial acetic acid, acetonitrile, and methanol (25 : 65 : 10); a column temperature of 35°C; injection volume 50 *μ*L and UV detection *λ* = 251 nm.

The sample to assess the total drug amount was prepared in the following steps: acetonitrile was added to the NLCs and sonicated for 30 minutes. After cooling, the volume was completed with the same solvent, an aliquot was centrifuged at 376 RCF for 15 min, and the supernatant was diluted with mobile phase to appropriate concentration. Aliquots of the NLC were filtered through Amicon Ultra-0.5 mL Ultracel-10 membrane 50 kDa centrifugal filters (Millipore Merck). The filter was checked for BPQ adsorption previously. The filtrate was directly injected to quantify the supernatant content. Both samples were analyzed by HPLC as described above (*n* = 3). Buparvaquone encapsulation efficiency was calculated by the difference between total BPQ amount and the content in the supernatant.

In the matter to test if the NLC structure could increase the drug loading, two formulations with augmented amount of BPQ were prepared, and their encapsulation efficiency was determined. A stability study was conducted at 8°C for three months; the samples were stored in 8 mL borosilicate sealed vials.

## 3. Results and Discussion

### 3.1. Evaluation of BPQ Solubility in Solid and Liquid Lipids

Table 1 shows the results of BPQ solubility in liquid lipids determined by HPLC. Miglyol® 182 presented the highest solubility (11.55 g·kg^−1^). This lipid is widely used for parenteral, oral, and topical formulations; it is resistant to oxidation and it is a synthesized medium-chain triglyceride, which ensures uniformity of product batch by batch.

**Figure 1:**
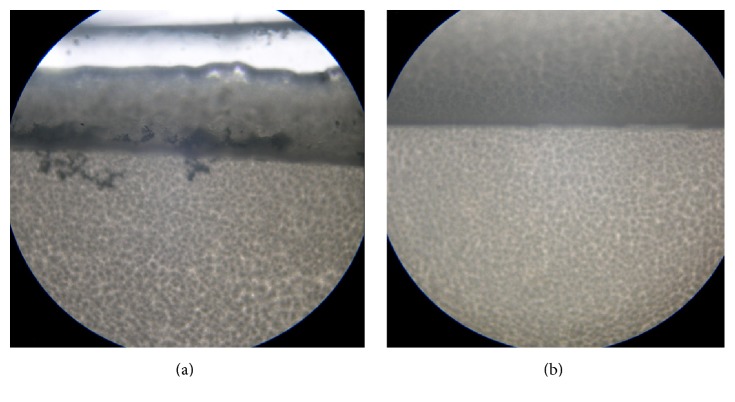
Optical microscopy from Softisan 154 lipid samples mixed with buparvaquone (a): (2 mg·g^−1^) (b): (1 mg·g^−1^). 40x magnification.

Table 2 shows the results for solid lipids using the microscopy method. The lipids were ranked by the likely presence of crystals. Figures 1(a) and 1(b) show the criteria for crystals observation in the mixture.  Figure 1(a) shows the lipid mixture, where BPQ was not soluble, and Figure 1(b) shows the lipid mixture, where BPQ was soluble.

Microscopic assessment for the determination of drug solubility in a solid lipid was used by several authors [24–29]. However, it is time-consuming; the results are semiquantitative and the interpretation can vary depending on the qualification of the analyst.

To find a potential substitute or an auxiliary method for microscopic evaluation, differential scanning calorimetry and thermogravimetry (DSC/TG) were used as a faster and reproducible approach for evaluating the ability of the solid lipids to solubilize BPQ. Kovács et al. [13], Severino et al. [30], and Liu et al. [31], in their studies, provided the rational for the correlation of DSC data with solubility. The greater the reduction in CI of a drug peak, in a physical mixture with the SL, the better the ability of the lipid to solubilize the drug.

**Figure 2:**
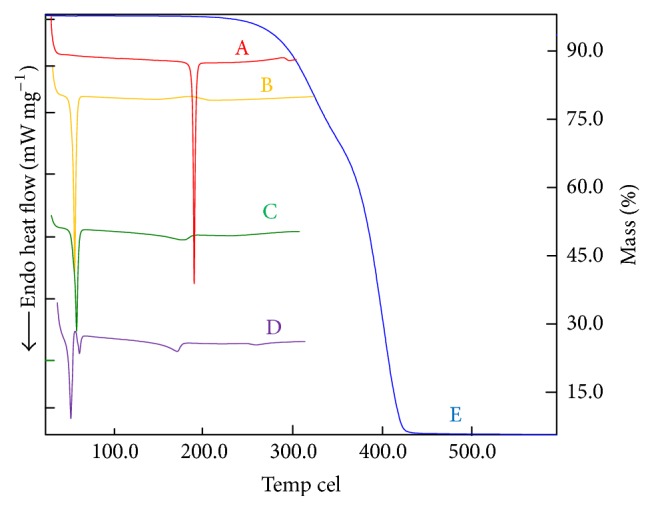
Thermoanalytical profiles of buparvaquone (BPQ), Softisan 154, and BPQ physical mixture (PM) obtained at 10°C min^−1^, under a dynamic nitrogen atmosphere (50 mL·min^−1^). BPQ DSC curve (A); DSC Softisan 154 curve (B); PM 1st run (C); PM 2nd run (D); TG: TG curves of PM (E). BPQ: buparvaquone; 1st run: first heat cycle; 2nd run: second heat cycle.

Table 3 shows CI of BPQ and SL mixtures.  Figure 2 shows Softisan 154 DSC/TG curves. For the other lipids, the same approach was performed. In the first heating cycle, the drug was more soluble in Gelucire 50/13 and Witepsol E85. The crystallinity index of BPQ was reduced to 40.6 and 48.8% for these lipids, respectively. In the second heating cycle, the Witepsol E85 and Softisan 154 showed almost the same CI, 49.0 and 49.1%, respectively. A second heating cycle was studied to simulate the manufacturing conditions of NLC by high-pressure homogenization [15, 26]. By quench-cooling the sample, the contact time between drug and molten lipid is increased, and it is possible to evaluate better the capacity of the lipid to solubilize the drug. Additionally, it is possible to evaluate the drug and lipid recrystallization behavior. This method can also provide information about drug degradation and or drug-lipid incompatibility.

Microscopy revealed that Softisan 154, among the other tested lipids, presented highest BPQ solubility. However, the DSC results confirmed its high solubility as the second rank, Witepsol being the first (Table 3). The divergence in the results revealed the relevance of the two methods.

By these findings, a rational for using the combined methods involves two steps: the screening of a high number of lipids with DSC/TG and microscopy evaluation for the most suitable ones to confirm and determine the drug amount that is soluble in the lipid. This combination of methods can save time, materials, and work labor and can generate important information about the drug and lipids.

### 3.2. Preparation of BPQ-NCL

The Softisan 154 (SL) and Miglyol 182 (LL) were mixed in different ratios to determine the maximum proportion of Miglyol 182. The SL : LL mixtures were visually homogeneous (no phase separation), and their melting points (MP) were above 40°C. For example, SL : LL 1.5 (3 : 2), the MP was 48.8°C. The SL : LL ratios where the mixtures showed melting points higher than 40°C were selected to ensure the NLC solid matrix structure [32].

**Figure 3:**
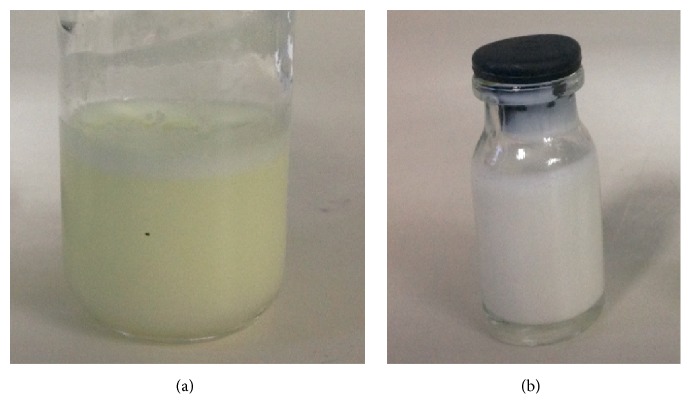
Nanostructured lipid carrier for buparvaquone encapsulation: (a) and (b), before and after high-pressure homogenization, respectively.

Figure 3 shows visual aspect of the formulations, before homogenization (Figure 3(a)) and after homogenization (Figure 3(b)). The high-pressure homogenization proved to be reliable for BPQ-NLC preparation; all formulations showed low *z*-averages <350 nm and low polydispersity <0.3 (Table 4). The yellow color of the drug was not observed after homogenization for F1 (Figure 3), indicating the drug internalization into the lipid matrix, which was confirmed by encapsulation efficiency. The stability of the formulations was accessed; all of them presented *z*-average and polydispersity variation less than 10% during three months (data now shown).

### 3.3. BPQ-NLC Optimization by Response Surface Design

The response surface methodology (RSM) is an efficient and powerful statistical method to optimize processes. Using the RSM, it is possible to reduce the number of experimental tests, with consequent savings in time and materials. The statistical design identifies component interactions and critical process parameters. The methodology involves three steps: (1) the development of rotary central composed design; (2) the response surface modeling by regression analysis (ANOVA); and (3) the optimization of the process using the model [33].

For this work, 20 experiments (Table 4) were used to evaluate the influence of the SL : LL and concentrations of Kolliphor P188 and Tween 80 in the *z*-average. The best fitting model from ANOVA was selected based on the comparison of statistical parameters including the *R*-square (*R*^2^), adjusted *R*-square (adj-*R*^2^), predicted *R*-square (pred-*R*^2^), lack-of-fit test, and *p* values. The linear model and its significance were revealed by the *p* value < 0.05 (*α* = 0.05) for each term (Table 5).

If the experimental environment is very noisy or significant variables are not considered in the experiment, it is possible that fraction of the observed data cannot be representative of the product/process. No significant lack-of-fit alone ensures model adequacy and therefore, measures of overall performance, referred to as *R*-squares (*R*^2^), were evaluated. For this work, *R*^2^ of 94.41%, adjusted *R*^2^ (adj-*R*^2^) of 92.92% and predicted *R*^2^ (pred-*R*^2^) of 91.04% show a suitable model from the data collected (Table 6).

When *R*^2^, adj-*R*^2^, and pred-*R*^2^ differ dramatically, there is an indication that nonsignificant terms were included in the model [34]. For the developed *z*-average model, the three *R*^2^ values are close (Table 6), which means the model is representative of the data and variables were representative of the product, regarding *z*-average.

Additionally, the pred-*R*^2^ indicates the high probability of the practical values being close to the theoretical calculations from the model. In the present study, the observed values were within the predicted range (95% CI) (Table 6). Therefore, the model can predict the *z*-average as a function of SL : LL ratio and surfactants concentrations. The *z*-average is a critical quality attribute for intravenous administration and macrophage uptake. It was found that nanoparticles between 100 and 300 nm are more appropriate for macrophage internalization by phagocytosis [35, 36]. Smaller particles (<100 nm) tend to be taken up by endocytosis, a process which occurs in virtually all cells

(2) Z=333.5+9.15SLLL−31.98T80−42.34K188+10.83T80∗K188,

where *Z* is the *Z*-average; SL : LL is the solid and liquid ratio; T80 is the % of Tween 80 (w/v); and K188 is the % of Kolliphor P188 (w/v).

**Figure 4:**
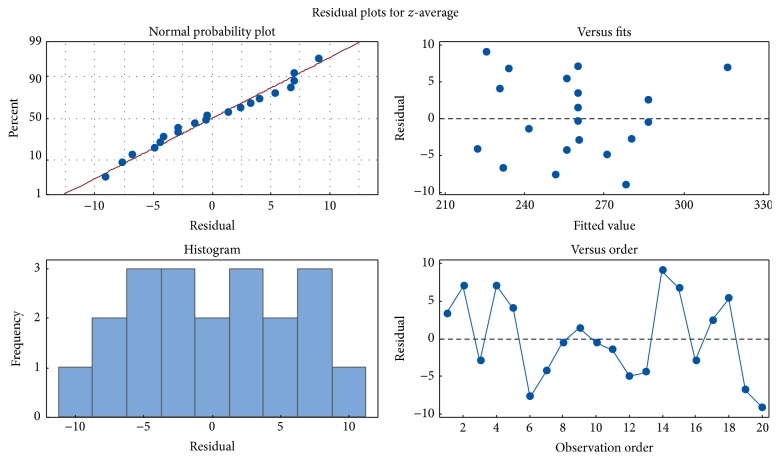
Residuals analysis of the BPQ-NLC mathematical model for *z*-average.

Residuals investigation is an essential part of all statistical modeling. The residual analysis for the ANOVA model was verified by the normal probability plot and the histogram (Figure 4). In normal probability plot, the residue is represented according to its expected value, calculated from the assumption that the residues follow a normal distribution [37]. Considering the results, the location of points on the probability curve, and the histogram, the normality assumption is valid. Also, the graph of the individual observations showed the random behavior of the residuals. There was no phenomenon of heteroscedasticity. The analysis of the residuals confirmed that the choice of this model was appropriate [37].

**Figure 5:**
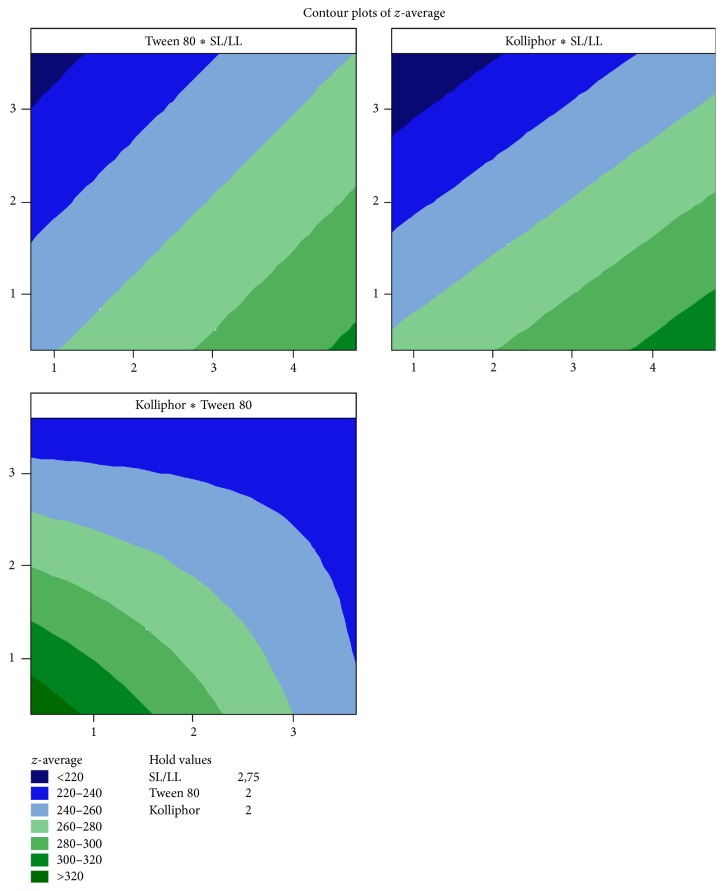
Contour plot of the BPQ-NLC mathematical model for *z*-average, containing the following variables: solid and liquid ratio (SL : LL), Kolliphor P188 and Tween 80. “*∗*” = versus.

Figure 5 showed that Kolliphor P188 reduced *z*-average at a lower concentration than Tween 80. The result showed the area of *z*-average <220 nm for Kolliphor P188 is larger than Tween 80. Likewise, the coded coefficient for Kolliphor P188 and Tween 80 is −33.78 and −16.85 (Table 6), which means that, in the same concentration, the formulation prepared using Kolliphor P188 will present lower *z*-average than the one prepared using Tween 80.

This finding can be explained by the longer central hydrophobic chain of polyoxypropylene from Kolliphor. Cohesive energy of lipophilic chain is stronger when compared to short lipophilic chain from Tween 80 [38, 39]. Moreover, two hydrophilic chains of polyoxyethylene oxide (PEO) exhibit gelation phenomena when heated [40], which contributes to integrity and stability of the surfactant film.

According to Figure 5, when adjusting the SL : LL ratio to 2.75 (11 : 4), surfactants above 3% (w/w) are required to reach low *z*-averages (see Table 7). The interaction between surfactants can be observed by the plot Kolliphor *∗* Tween and by the Kolliphor *∗* Tween coefficient interaction term, which presents a positive value of +28.88 (Table 6). As a consequence, for the optimization, low amounts of Tween 80 were applied to reduce the impact of this interaction. Furthermore, it can be seen that *z*-average <220 nm are only achieved at low ratios of SL : LL (<2.5).

**Figure 6:**
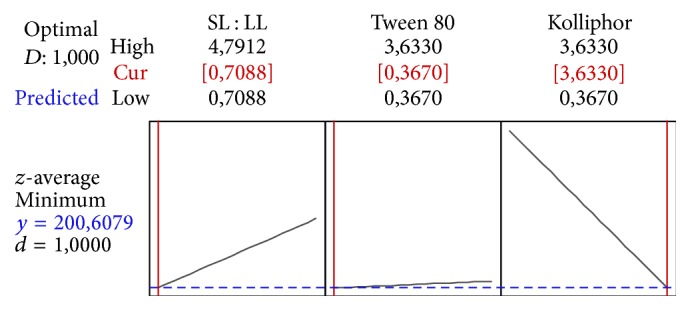
Profile for predicted values and desirability function of the F1 *z*-average (201 nm), containing the following variables: solid and liquid lipid ratio (SL : LL) at 0.71, Kolliphor P188 at 3.6% (w/w), Tween 80 at 0.37% (w/w).

**Figure 7:**
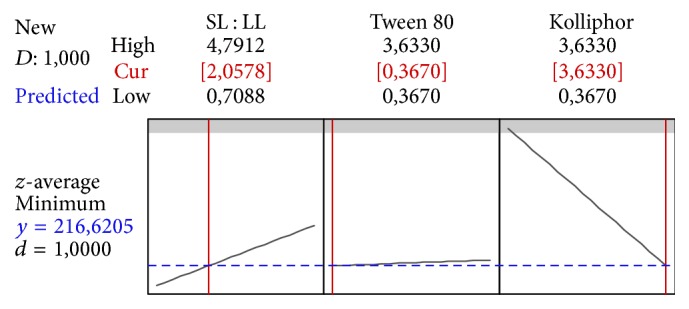
Profile for predicted values and desirability function of the F2 *z*-average (217 nm), containing the following variables: solid and liquid lipid ratio (SL : LL) at 2.06, Kolliphor P188 at 3.6% (w/w), Tween 80 at 0.37% (w/w).

For the mathematical model confirmation, two formulations were prepared: F1 and F2, according to Figures 6 and 7, respectively. The measured values of *z*-average for F1 were 215.4 nm ± 1.7% (183.8 to 217.4 nm; CI 95%). For F2, they were 231.2 nm ± 1.0% (200.8 to 232.4 nm; CI 95%), which are within the predicted range (Table 6), confirming the validity of the mathematical model.

### 3.4. Encapsulation Efficiency (EE)

Table 8 shows the BPQ EEs for three formulations containing 320 (F1), 400 (F3), and 500 *μ*g·mL^−1^ (F4) of BPQ. These formulations were prepared using the same SL : LL ratio and surfactant concentration. They were prepared aiming to increase the drug loading since the structure of the matrix is disorganized by the presence of the liquid lipid and more drugs could be accommodated, as reported by Chinsriwongkul et al. [41] and Yuan et al. [42]. For these three formulations, the EEs were close to 100%, even in the F4, which was prepared with 156% of calculated drug loading. At the end of 3 months of stability, for all formulations, no drug was precipitated and no significant changes in the EE were found (data not shown).

These findings revealed the role of NLCs for BPQ encapsulation, comparing to SLN and NE. The preparation of SLN would compromise the drug loading and encapsulation efficiency during stability since BPQ has low solubility in solid lipids. The development of NE could increase the drug loading for using only liquid lipid. However, the stability of the nanoparticles and the increasing amount of surfactants, or even a required replacement for more sophisticated and expensive surfactants, such as lecithin, would lead to complex formulations and, consequently, expensive medicines, which is a big concern for neglected diseases.

## 4. Conclusions

DSC/TG method revealed BPQ best solubility using Witepsol E85 and Softisan 154 as solid lipid. Microscopy supported Softisan 154 suitability for NLC preparation. Both techniques, in combination, proved to be a powerful and consistent tool to select the appropriate solid lipids in the preformulation studies. The Miglyol 182 allowed a drug loading up to 0.5 mg/mL due to the high solubility of BPQ (11.55 g·kg^−1^) in this liquid lipid.

The response surface methodology identified the critical surfactants concentration and Softisan 154 and Miglyol 812 ratios and the interaction between Kolliphor P188 and Tween 80. The relationship among these variables, demonstrated by a linear mathematical model, allowed generating a design space. This design space revealed the limits in the variables for the achievement of *z*-averages at submicron size range.

The developed nanostructured lipid carriers showed low *z*-averages (<350 nm), polydispersity (<0.3), and encapsulation efficiency close to 100%, even when the BPQ amount was raised 156%, endorsing the ability of the NCL to increase the drug loading of lipid-based nanoparticles.

Thus, these findings showed the successful development of buparvaquone nanostructured lipid carriers, which are promising formulations for the treatment of leishmaniases. This drug delivery system has the potential to improve the availability of affordable medicines due to the low cost of raw materials, using technology with well-established reliability, efficacy, and scale-up feasibility.

## Figures and Tables

**Table 1:** Buparvaquone solubility in liquid lipids.

Liquid lipid	Solubility (g·kg^−1^)	RSD (%)
Miglyol 182	11.55	1.9
Glyceryl monocaprylate	7.43	1.3
Safflower purified oil	6.48	1.6
Corn purified oil	6.51	0.6
Olive purified oil	6.21	1.6
Sesame purified oil	5.96	7.4
Cottonseed purified oil	6.01	0.5
Soy purified oil	5.43	7.3
Oleic acid	5.21	2.4

**Table 2:** Crystal evaluation by microscopy for buparvaquone solubility test.

Crystals rank	Lipid	Soluble at 1 mg·g^−1^	Soluble at 2 mg·g^−1^	Melting point (°C)
1st	Softisan 154	Yes	No	53–58
2nd	Gelucire 44/14	No	No	44
3rd	Dynasan P60	No	No	58–62
4th	Compritol 888	No	No	70
5th	Dynasan 118	No	No	72–75
6th	Sterotex HM	No	No	69
7th	Witepsol E85	No	No	42–44
8th	Gelucire 50/13	No	No	50
9th	Precirol ATO 5	No	No	56

**Table 3:** Enthalpy of fusion (J·mg^−1^) and crystallinity index for determining the solubility of buparvaquone in solids lipids by differential scanning calorimetry.

Lipid	Δ*H*_BPQ_	Δ*H*_BPQ-PM_	Δ*H*_BPQ-PM_	CI (%)	CI (%)
	(J·mg^−1^)	(J·mg^−1^) 1st	(J·mg^−1^) 2nd	1st	2nd
Witepsol E85	136.0	33.2	33.3	48.8	49.0
Softisan 154	136.0	42.2	33.4	62.1	49.1
Gelucire 50/13	136.0	59.4	34.4	40.6	50.6
Gelucire 44/14	136.0	47.6	35.5	70.0	52.2
Precirol ATO 5	136.0	54.9	37.7	80.7	55.4
Sterotex HM	136.0	51.5	44.0	75.7	64.7
Dynsan P60	136.0	59.4	57.9	87.4	85.1
Compritol 888	136.0	66.5	67.3	97.8	99.0

BPQ: buparvaquone; BPQ-PM: BPQ and lipid physical mixture; CI: crystallinity index = (BPQ enthalpy in lipid mixture (J·mg^−1^)*∗D* (proportion of BPQ and lipid)/BPQ enthalpy of fusion (J·mg^−1^)) × 100; 1st and 2nd heating cycles.

**Table 4:** Experimental matrix and values of *z*-average and polydispersity (PDI) of buparvaquone nanostructured lipid carriers.

Form	Order	SL : LL	% Tween 80 (w/w)	% Kolliphor P188 (w/w)	*z*-average (nm)	PDI
12	1	2.75	2.00	2.00	263.2	0.198
2	2	4.00	1.00	1.00	323.1	0.252
6	3	4.00	1.00	3.00	257.3	0.214
9	4	2.75	2.00	2.00	266.9	0.194
5	5	1.50	1.00	3.00	234.6	0.154
8	6	4.00	3.00	3.00	243.9	0.228
7	7	1.50	3.00	3.00	217.7	0.210
1	8	1.50	1.00	1.00	286.0	0.193
11	9	2.75	2.00	2.00	261.3	0.224
10	10	2.75	2.00	2.00	259.4	0.207
3	11	1.50	3.00	1.00	239.7	0.179
4	12	4.00	3.00	1.00	265.9	0.190
20	13	2.75	2.00	2.00	251.4	0.192
18	14	2.75	2.00	3.63	234.2	0.193
16	15	2.75	3.63	2.00	240.5	0.220
14	16	4.79	2.00	2.00	277.2	0.212
17	17	2.75	2.00	0.37	289.0	0.178
19	18	2.75	2.00	2.00	261.2	0.198
13	19	0.71	2.00	2.00	224.8	0.162
15	20	2.75	0.37	2.00	268.8	0.157

Form: formulation; order: preparation order; SL : LL: solid and liquid lipid ratio.

**Table 5:** Analysis of variance for *z*-average.

Source of variation	DF	SS (adj)	MS (adj)	*F*-value	*p* value
Model	4	9807.0	2451.74	63.33	0.000
Linear	3	8868.9	2956.29	76.36	0.000
SL : LL	1	1744.6	1744.56	45.06	0.000
Tween 80	1	1419.4	1419.38	36.66	0.000
Kolliphor P188	1	5704.9	5704.95	147.36	0.000
Tween 80^*∗*^Kolliphor P188	1	938.1	938.09	24.23	0.000
Error	15	580.7	38.71		
Lack-of-fit	10	274.6	27.46	0.45	0.869
Pure error	5	306.1	61.22	—	—
Total SS	19	10387.7			

DF: degrees of freedom; SS: sum of squares; *F*-value: statistic *F*-test; MS (adj) adjusted mean square; *p* value: statistical significance. SL : LL: solid and liquid ratio. SD = 6.22210; *R*^2^ = 94.41%; adj-*R*^2^ = 92.92%; pred-*R*^2^ = 91.04%.

**Table 6:** Coded coefficients of the linear model and *p* value (*α* = 0.05) with the variables: liquid and solid lipid ratio (SL : LL), Kolliphor P188 (K188), and Tween 80 (T80).

Terms	Coefficient	*C*- DP	*T*-value	*p* value	VIF
Constant	253.30	1.39	182.06	0.000	
SL : LL	18.68	2.78	6.71	0.000	1.00
T80	−16.85	2.78	−6.05	0.000	1.00
KP188	−33.78	2.78	−12.14	0.000	1.00
T80 *∗* K188	28.88	5.87	4.92	0.000	1.00

**Table 7:** Observed and predicted *z*-average, polydispersity index (PDI), and zeta potential (ZP) results from buparvaquone nanostructured lipid carriers.

	Observed *z*-average (nm) ± RSD (%)	Predicted *z*-average (nm) (*α* = 0.05)	PDI ± RSD (%)	ZP (mV) ± RSD (%)
F1	215.4 ± 1.7	183.8 to 217.4	0.152 ± 7.6	−20.3 ± 4.7
F2	231.2 ± 1.0	200.8 to 232.4	0.136 ± 4.9	−20.9 ± 1.0

F1: SL : LL: 0.71, Tween 80: 0.37% (w/w), and Kolliphor P188: 3.63% (w/w); F2: SL : LL: 2.06, Tween 80: 0.37% (w/w), and Kolliphor P188: 3.63% (w/w).

**Table 8:** Theoretical and observed drug load and encapsulation efficiency of buparvaquone nanostructured lipid carriers.

	BPQ theoretical concentration (*μ*g·mL^−1^) (*n* = 3)	Total observed BPQ concentration (*μ*g·mL^−1^)	Supernatant BPQ (*μ*g·mL^−1^)	Encapsulation efficiency (%)
F1	320	312.5 ± 1.4	0.13 ± 0.02	99.96 ± 0.06%
F3	400	403.57 ± 5.0	<limit of detection	100.00%
F4	500	477.87 ± 1.9	0.06 ± 0.01	99.99 ± 0.02%
